# Is prone free breathing better than supine deep inspiration breath-hold for left whole-breast radiotherapy? A dosimetric analysis

**DOI:** 10.1007/s00066-020-01731-8

**Published:** 2021-01-08

**Authors:** Xinzhuo Wang, Odile Fargier-Bochaton, Giovanna Dipasquale, Mohamed Laouiti, Melpomeni Kountouri, Olena Gorobets, Nam P. Nguyen, Raymond Miralbell, Vincent Vinh-Hung

**Affiliations:** 1 Radiation Oncology, Tianjin Union Medical Center, 300121 Tianjin, China; 2 Radiation Oncology Department, Geneva University Hospital, Geneva, Switzerland; 3 Service de radio-oncologie, Hôpital Riviera-Chablais, Rennaz, Switzerland; 4 CHU de Martinique, Fort-de-France, Martinique, France; 5 Howard University, Washington DC, USA; 6 Proton Therapy Centre, Quirónsalud, Madrid, Spain; 7 Institut Oncològic Teknon (IOT), Barcelona, Spain

**Keywords:** Linear models, Cardiotoxicity prevention, Radiation dosage, Mean absolute dose deviation, Weighted excess dose deviation score, Dose volume histogram

## Abstract

**Purpose:**

The advantage of prone setup compared with supine for left-breast radiotherapy is controversial. We evaluate the dosimetric gain of prone setup and aim to identify predictors of the gain.

**Methods:**

Left-sided breast cancer patients who had dual computed tomography (CT) planning in prone free breathing (FB) and supine deep inspiration breath-hold (DiBH) were retrospectively identified. Radiation doses to heart, lungs, breasts, and tumor bed were evaluated using the recently developed mean absolute dose deviation (MADD). MADD measures how widely the dose delivered to a structure deviates from a reference dose specified for the structure. A penalty score was computed for every treatment plan as a weighted sum of the MADDs normalized to the breast prescribed dose. Changes in penalty scores when switching from supine to prone were assessed by paired *t-*tests and by the number of patients with a reduction of the penalty score (i.e., gain). Robust linear regression and fractional polynomials were used to correlate patients’ characteristics and their respective penalty scores.

**Results:**

Among 116 patients identified with dual CT planning, the prone setup, compared with supine, was associated with a dosimetric gain in 72 (62.1%, 95% CI: 52.6–70.9%). The most significant predictors of a gain with the prone setup were the breast depth prone/supine ratio (>1.6), breast depth difference (>31 mm), prone breast depth (>77 mm), and breast volume (>282 mL).

**Conclusion:**

Prone compared with supine DiBH was associated with a dosimetric gain in 62.1% of our left-sided breast cancer patients. High pendulousness and moderately large breast predicted for the gain.

## Introduction

Breast cancer is the most commonly diagnosed cancer and the leading cause of cancer death in women worldwide [1]. Death rates have been stable so far or slightly declining in some countries [2, 3], which may reflect early detection and/or improved treatment. In order to further improve survival and to reduce the risk of treatment sequelae, more clinical research is needed. Randomized trials have shown the importance of radiotherapy for optimal local control of breast cancer [4]. Yet, despite a 67% reduction in local recurrence rates, the survival benefit for those patients treated with postoperative radiotherapy has been disproportionately modest [4]. There has been concern that local control is offset by an increased risk of heart, vascular, and lung toxicity [5–14]. For decades, one of the major challenges facing radiation oncologists has been to reduce the risk of toxicity without decreasing the chances of cancer control and survival [10]. A good number of technical procedures seeking to find the best trade-off between side effects and tumor control are actively pursued [15–20]. This subject matter is even more relevant when considering treatment optimization for left-sided breast cancer [21–28]. A prone setup has been advocated to spare the left lung and the heart when irradiating such patients. However, most published studies addressing this question have limited their scope to large breasts only [26, 29] (mean 896 mL in an on-going review of 21 studies 2007–2015, V. Vinh-Hung). With a prone setup, the breast sags from the chest wall, allowing tangential fields to avoid the heart and the left lung. However, the dose to the heart might increase due to movement of the heart anteriorly in the prone position [30]. The dosimetric implications of prone positioning therefore will depend on the location of the breast target tissues relatively to the heart and chest wall [31].

Considering the variability of anatomic characteristics, organ displacement, and postsurgical changes, one may need customized planning comparisons to be able to select between prone and supine for every patient. Drawbacks are two computed tomography (CT) simulations and twice the dosimetry, the dose burden of double CT exposure to the patients, and an increased workload for oncologists and dosimetrists [32–34]. Clinical tools are needed to determine beforehand which position would be most advantageous.

Since 2010 through 2013, dual planning has been applied in our institution for most patients who had been referred for adjuvant radiotherapy after conservative surgery. In the present study, we evaluated left-sided breast cancer patients simulated both prone in free breathing and supine under deep inspiration breath-hold (DiBH) conditions. We aimed to investigate whether a change in the treatment position setup from supine to prone was associated with a dosimetric gain for these left-breast cancer patients, and to identify characteristics correlated with the gain.

## Materials and methods

Patients were retrospectively retrieved from the Geneva University Hospital radiation oncology department database. We selected patients with left-sided breast cancer referred from September 2010 to August 2013 for adjuvant radiotherapy after conservative surgery with completely resected primary breast cancer. All selected patients underwent dual CT simulation and treatment planning, prone in free breathing and supine in DiBH conditions. Patients gave their written consent prior to simulation. Double CT simulation was not done if the patient expressed discomfort during the prone setup after giving their consent. The study received institutional review board approval and was registered under ClinicalTrials.gov Identifier NCT02237469.

In the supine setup patients were positioned on an inclined breast board with arms extended over the head [35, 36]. For the prone setup patients were positioned using the Bionix Prone Breast System (Bionix Development Corporation, Toledo, OH, USA) (2010–2012) and the Varian Pivotal Prone Breast Care (Varian Biomedical Systems, Palo Alto, CA, USA) from 2013. Covering both lungs and breasts from the top of the lungs to 5 cm caudal to the breasts or to the base of the lungs, whichever was the most caudal, 3‑mm thick CT slices without contrast were used. The right breast rested on a 5-degree foam wedge. The left breast was intended to hang centered and unhindered through the couch’s opening. A patient self-assessed questionnaire recorded the patient’s subjective feelings of pain, fear, anxiety, discomfort, and position preference at the end of simulation [37].

The breast clinical target volume (CTV) was contoured up to 1 cm below the sternoclavicular joint (cranially), to the farthest visible breast contour (caudally), to the perforating mammary vessels or to the edge of the sternum (medially), to the lateral breast-skin fold (laterally), to, but not beyond, the surface of the pectoralis muscle or ribs and intercostal muscles (posteriorly), and up to 5 mm under the skin surface (anteriorly) [38]. The tumor bed CTV was based on combined clinical, radiological, and surgical-pathological data. The planning target volume (PTV) equated to the CTV without expansion. Contouring the contralateral breast included the skin surface; contouring the heart included the pericardium and the exiting large vessels [39], but not above the top of the left atrium. Automatic segmentation contoured both the lungs and the body’s external contour.

The dose prescription for most patients was 47.25 Gy to the breast in 21 fractions, 4 fractions/week [40]. Treatments were planned with tangential fields using the Varian Eclipse treatment planning system with the prescription of 95% of the dose covering at least 95% of the breast PTV, covering 100% of the tumor bed, and the breast PTV V_107%_ <2 cc. Dose constraints to the organs at risk (OAR) were the following: ipsilateral lung V_20_ _Gy_ <10%, heart near max D_2%_ <15 Gy, and heart mean dose <3 Gy. Beam arrangements were required to avoid the contralateral breast. The chest wall was excluded from the PTV. Planning implemented forward intensity-modulated radiotherapy [41], combining wedges, field-in-field compensation, and a mix of photon energies. Heart shielding was undertaken if necessary using a multileaf collimator [42]. All doses were converted to percent values of the prescribed dose.

A penalty score was built from the mean absolute dose deviation (MADD) [43]. The MADD of a structure measures how far the planning dose deviates from a given reference dose specified for the structure. Applying the notation *M*_*i*_ as the MADD of structure *i, D* the dose abscissa and *V* the volume ordinate of the set of points representing the cumulative dose–volume histogram (DVH) curve of the structure, *V*_0_ the volume of the structure, and *A *the reference dose for the structure (*D, V, V*_0_, and *A* subscript *i* implicit), the MADD *M*_*i*_ can be defined as

$$M_{i}=\int _{0}^{V_{0}}\frac{\left| D-\right.\left.A\right| }{V_{0}} dV.$$

**Fig. 1:**
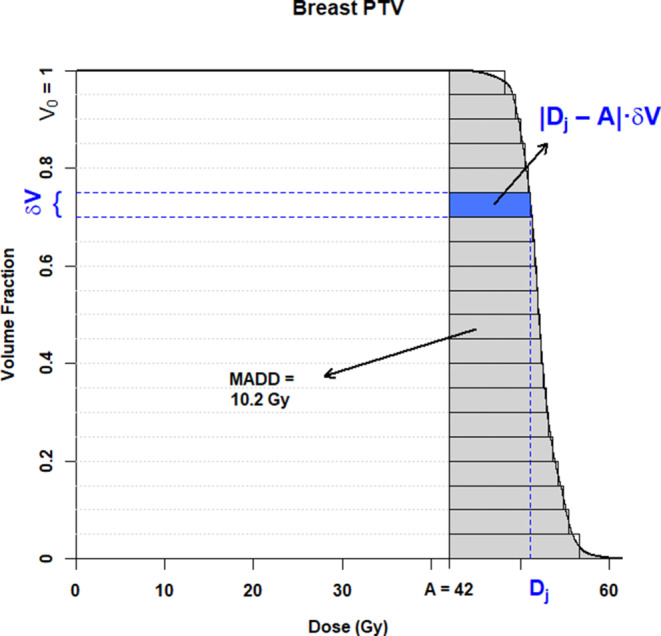
Computation of the mean absolute dose deviation (*MADD*). Dose–volume histogram (DVH) of a fictive case prescribed 42 Gy to the breast planning target volume (*PTV*). The MADD is the *grey shaded area* between the DVH curve and the prescription dose, *A* = 42 Gy. At each point *j* of the DVH, the deviation is |*D*_*j*_ *−* *A*|. The deviation on a *δV* volume interval is the *horizontal rectangle* between the curve and the reference, *shaded blue* at the instance of the dose *D*_*j*_. The total *grey area* is approximated by the sum of the plain rectangles. If the DVH has been plotted with the volumes already scaled to 1 as in this figure, *V*_0_ = 1, the area directly provides the MADD, otherwise the area needs to be divided by the structure’s volume

The equation represents the area *between* the DVH of the structure and the reference dose *A*, where *A *is 0 for an OAR, or the prescribed dose for a PTV. Graphically, the MADD *M*_*i*_ is a *horizontal* integration with respect to *V*. Computation can be implemented by rectangular strips (Fig. 1), by writing for a set of *n* DVH datapoints

$$M_{i}=\sum _{j=1}^{n}\frac{\left| D_{j}-A\right| \times \delta V}{V_{0}}.$$

The difference |*D*_*j*_ *−* *A*| is a difference between doses, *δV /V*_0_ cancel out as unitless; hence, the MADD unit is the same as that used for the DVH, either in absolute or relative dose. The lowest theoretical *M*_*i*_ value is 0 for a perfect dose distribution.

The penalty score pertaining to a patient’s treatment plan was defined as a weighted sum of the MADDs:

$$\textit{Penalty}\,\textit{Score}=\sum _{i}w_{i}\times M_{i}$$

where *i* represents a list of structures, *M*_*i*_ represent the corresponding MADDs, and *w*_*i*_ represent the weights attributed to the structures. Constraining the weights to sum to 1, $\sum _{i}w_{i}=1$, allows expression of the penalty score on the same scale as the MADDs and the dose prescription. Unlike the homogeneity index which is a unitless ratio applicable only to target volumes, the penalty score so defined is interpretable as a weighted average of all OAR and PTV dose deviations.

The *w*_*i*_ weights selected by first intention in this study were as follows: 0.40, 0.16, 0.14, 0.11, 0.10, and 0.09, for the heart, right lung, left lung, tumor bed, right breast, and left breast, respectively. That set of weights, which we call “penalty type 1,” was chosen to represent the authors’ practice for breast cancer when considered as low risk. The motivation stems from the legacy of studies that investigated the impact of breast radiotherapy on mortality [4–13]. A set of ordinal priorities was established assigning heart > lungs > CTV tumor bed > contralateral breast > CTV ipsilateral breast, which were then converted to the numerical weights. Other priority types will be discussed later as an expanded study.

The prone penalty scores were compared with the supine penalty scores. The prone setup was considered to provide a dosimetric gain if it reduced the penalty. The comparisons were performed using the following three assessments: by aggregate comparison of the mean penalty scores using a *t*-test [44]; by assessing the number of patients in whom the prone setup provided a reduction in the penalty score, and computing the proportion’s confidence interval using the exact binomial test [45]; by visual comparison using a bullet-arrow graph that we designed as a generic tool to explore changes from a baseline. Briefly, the bullet-arrow graph procedure is as follows: 1) the patients’ supine penalty scores are sorted; 2) the sorted supine penalties are plotted as bullets; 3) the patients’ prone penalties are plotted as arrowheads; 4) the bullet and arrowhead pairs are joined—the resulting arrow segments indicate the magnitude and the direction of the penalty changes.

**Fig. 2:**
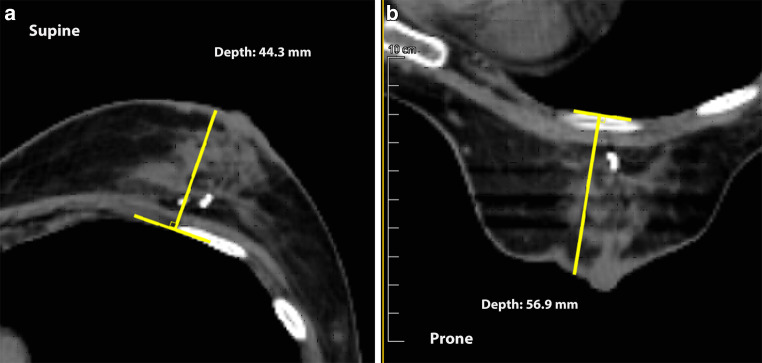
Measurement of breast depth supine (**a**) and prone (**b**). The depth was measured on the CT slice through the nipple using an on-screen adjustable T‑square ruler, recording the largest distance from the breast contour to the base set tangent to the pleura. If the breast point falls on the nipple, the measurement was performed flush with the areola

Two regressions were applied to evaluate the pre-dosimetry patient characteristics as potential predictors of a dosimetric gain: fractional polynomial regression to assess nonlinearity [46], and robust linear regression [47] if linearity was acceptable. The patient characteristics evaluated were as follows: age, height, weight, body mass index (BMI), tumor location, breast volume, patient’s setup preference, and CT-based distance between the left anterior descending coronary artery (LAD) and the chest wall [31], and the supine and prone breast depths (Fig. 2). Non-numeric characteristics were dummy binary coded as “0” for the reference and “1” for the other levels; regression measures the change in the response when the characteristic changes by one level [48]. The post-dosimetry values of the penalty scores were also assessed as additional predictors of the prone penalty score reduction.

All statistical computations used R version 3.6.3 [49]. The regressions were implemented using the package mfp with two degrees of freedom [46], and using function rlm of the package MASS [47]. Graphic displays used ggplot [50].

## Results

We identified 299 dual prone–supine breast CT simulation cases. Three cases were excluded—one volumetric modulated arc therapy and two non-finalized prone planning. Of the remaining 296 dual plans, 151 were right-breast treatments, leaving a total of 145 left-breast cases to be assessed, 27 of whom were unable to hold breath on supine at CT and were not eligible; 2 were repeat CT after initial simulation and were excluded. Thus, the total study population was composed of 116 patients with dual planning at initial simulation. Those included 2 patients with breast implants and 3 with bilateral cancer who had separate planning for the left and right breasts.

The median age of the patients was 57.5 years, range 36–82 (Table 1), 4.5 years younger than US patients [2] but comparable to another registry data [12]. Overweight and obesity were frequent and represented 51.5% of the non-missing records. The left breast median volume supine was 484 mL (range 34–1580) comparable to the median volume prone of 477 mL. The contralateral breast median volume supine was 597 mL (range 81–2138), prone 591 mL (range 88–1736). The patients preferred the supine setup in 52.6% of the cases, while 47.4% preferred prone or were indifferent.

**Fig. 3:**
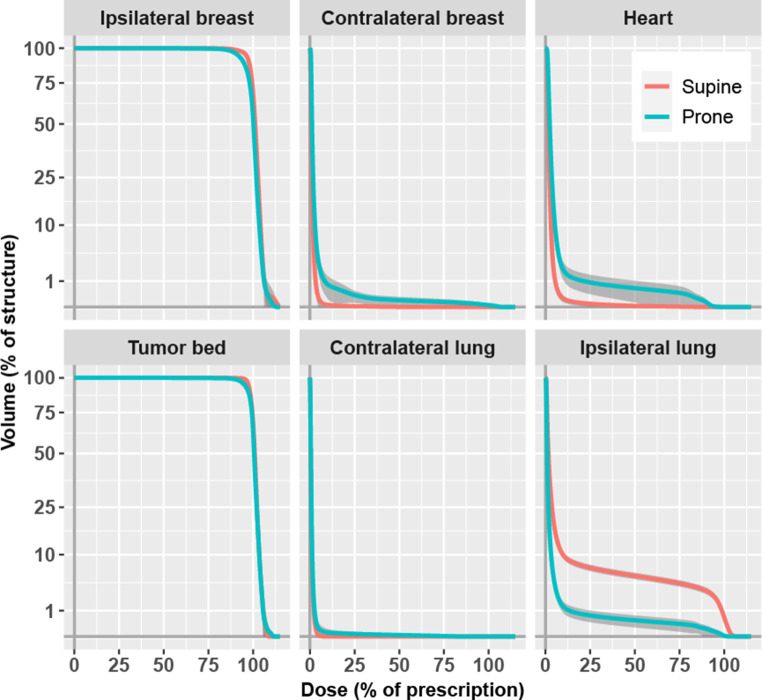
Averaged cumulative dose–volume histograms by structure and setup. Volume y‑axis square root scale. *Dark grey*: 99% confidence

Fig. 3 shows the cumulative DVHs of the six structures as the pointwise average for all 116 patients according to position setup. Compared to supine, the prone setup showed a small underdosage of the ipsilateral breast almost complying with the prescription dose, a higher dose to the contralateral breast, a higher dose to the heart, a slight underdosage to the tumor bed near the prescription dose, and a large reduction of the dose to the ipsilateral lung.

**Fig. 4:**
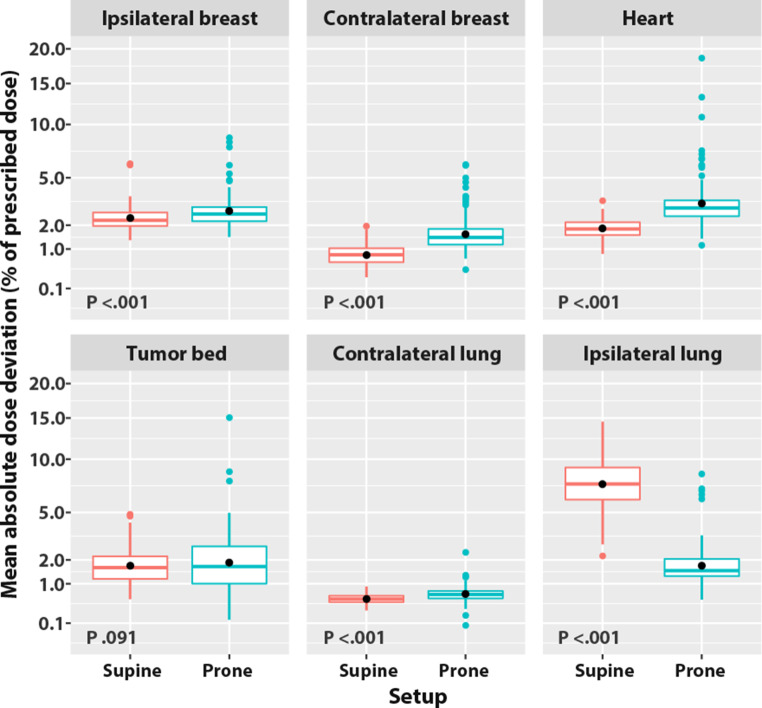
Mean absolute dose deviation (MADD) by structure and according to setup. MADD y‑axis square root scale. *Box*: lower quartile, median, upper quartile. *Whiskers*: 1.5 × interquartile range. *Black dot*: average of the MADDs. *Color dots*: outliers

The corresponding MADDs for the prone vs. supine setup are displayed in Fig. 4. All *p*-values were <0.001, except for that of the tumor bed, *p* = 0.091. The prone setup slightly increased (by a difference of <0.5% of prescribed dose) the MADDs of the ipsilateral breast, the tumor bed, and the contralateral lung. The prone setup increased more notably (by a difference of ≥0.5% of prescribed dose) the MADD of the contralateral breast, 1.7% of prescribed dose vs. that of the supine setup at 0.8%, and the MADD of the heart, 3.4% vs. 1.9% for the supine setup. The prone setup reduced the MADD of the ipsilateral lung by over two thirds, 1.9% vs. 7.6% for the supine setup.

Computation of the patients’ type 1 penalty scores found a mean penalty of 2.39% of prescribed dose in the prone setup as compared with 2.41% in the supine setup (two-sided *t*-test, *p* = 0.868). The median penalty score for prone was 2.14 (range 0.98–10.78), vs. 2.36 (range 1.33–4.26) for supine; the median penalty score change was −0.21 (range −2.47–7.24).

**Fig. 5:**
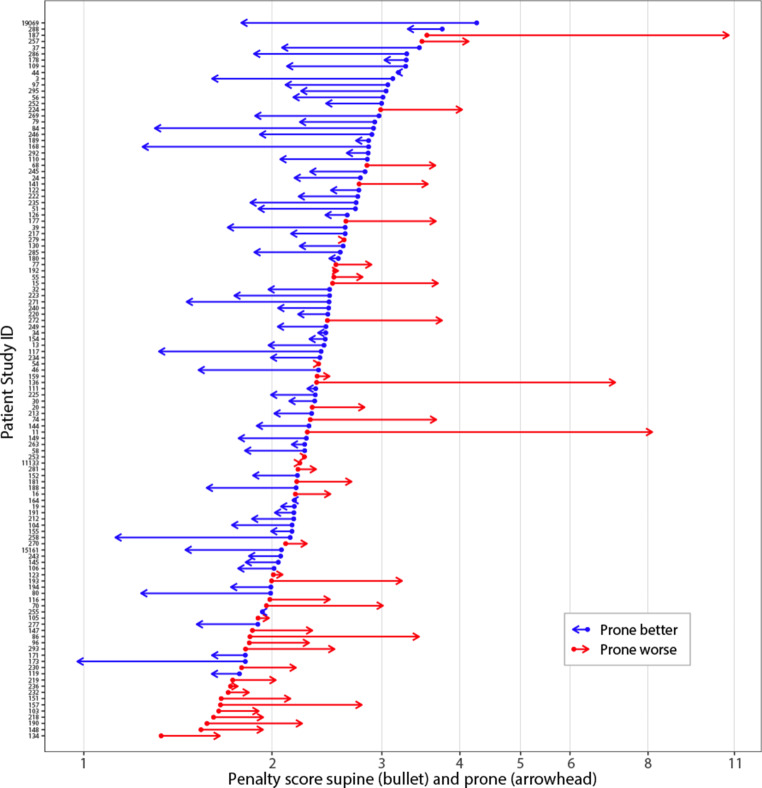
Patients’ prone penalty vs. supine penalty (type 1). Penalty score units are the percentages of the prescribed dose. *Bullet (dot)*: supine penalty. *Arrow*: prone penalty. *Red*: the prone setup increases the penalty, *blue*: the prone setup decreases the penalty

Fig. 5 displays that the penalty score was reduced for prone in 72 patients out of 116 (62.1%, 95% CI: 52.6%, 70.9%). The reduction was dependent on the value of the supine penalty: the prone setup reduced the penalty in 7 of 26 patients whose supine penalty was <2 (26.9%; 95% CI: 11.6%, 47.8%, lower third of Fig. 5) compared with a reduction in 65 of 90 patients whose supine penalty was ≥2 (72.2%; 95% CI: 61.8%, 81.1%; upper two thirds of Fig. 5), *p* < 0.001.

**Fig. 6:**
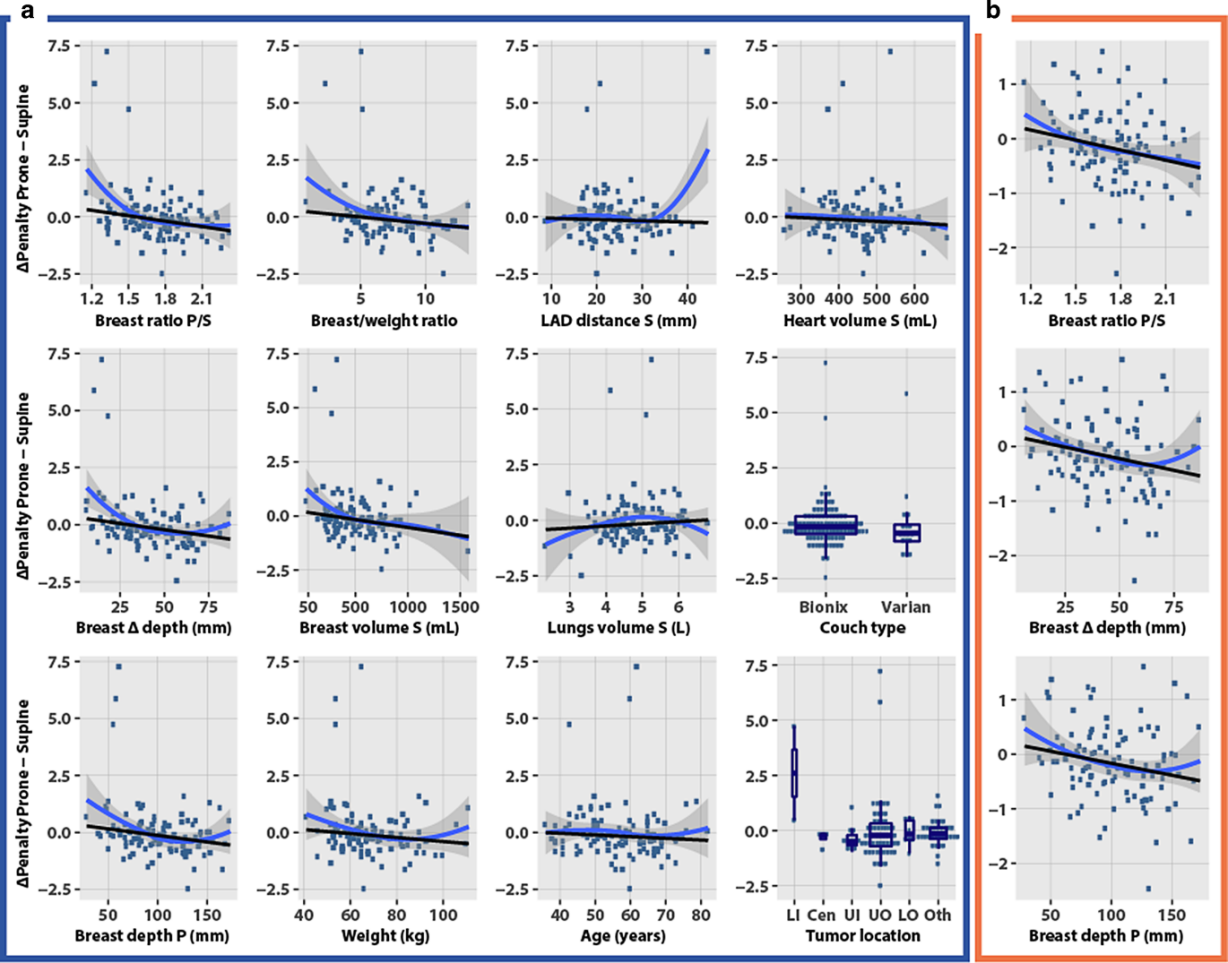
Predictors of the penalty (type 1) difference between prone and supine setups. Y‑axis, penalty score prone minus penalty score supine; positive, prone worse; negative, prone better. *Blue curve*: local polynomial smoothing and 95% confidence (descriptive); note the LAD curvature by a single outlier. *Black line*: robust linear regression. *P* prone, *S* supine, *LAD* left anterior descending artery, *LI* lower inner, *Cen* central, *UI* upper inner, *UO* upper outer, *LO* lower outer; *Oth* other. **a** All 116 cases. **b** Selected subplots excluding three outliers with ∆penalty >2.5, *N* = 113

Fig. 6a plots the penalty score differences (∆, prone minus supine) according to 12 selected characteristics. A positive y‑axis value indicates that the prone setup increased the penalty (prone worse) and a negative value indicates that prone reduced the penalty (prone better). Pendulousness indicators, breast ratio prone/supine, breast depth, breast ∆ depth prone − supine difference, and weight were associated with decreased penalty scores in the prone setup. Age, heart volume, and LAD–chest wall distance showed no strong correlation with ∆ penalty. Among the categorical characteristics, a trend favored the prone setup using the Varian couch. According to tumor location, the lower inner quadrant was associated with an increased prone penalty though this location was underrepresented with only 2 patients.

Three top outliers were apparent in the 12 subplots of Fig. 6a, corresponding to the 3 patients with the largest increase in the prone penalty in Fig. 5. Excluding these three outliers did not remarkably change the linear fit (Fig. 6b; only the first column of subplots is shown to avoid redundancy).

Table 2 based on the full patient data summarizes the models of dosimetric gain as a function of the patients’ characteristics. All the characteristics related linearly with the dosimetric gain, except the breast depth ratio, the breast ∆ depth difference, and the right breast volume for which fractional polynomial regression indicated a significant nonlinear functional form. Nonlinear transforms caused small differences in the estimates (Table 2 footnote). We retained only the linear regression models. The models were ranked into broad categories. The most significant pre-dosimetry predictors were the three indexes of breast pendulousness, the ratio of the breast volume to body weight, the lower inner quadrant tumor location, and the breast volume. Breast depth in supine setup and weight were borderline significant, *p* = 0.061 and *p* = 0.063, respectively. Quality indicators of DiBH such as larger lung volume expansion or LAD-to-chest wall distance, younger age, and patient’s preference were not significant.

**Table 1:** Patient characteristics

Characteristic	*N*	Value	Percent
*Age (years)*	116	–	––
Median (range)	–	57.5 (36–82)	–
*Pathological stage*	116	–	–
0	–	7	6.0
I	–	69	59.5
II	–	39	33.6
III	–	1	0.9
*pT*	116	–	–
Tis	–	7	6.0
T1	–	80	69.0
T2	–	28	24.1
T3	–	1	0.9
*Lymph node ratio (fraction)*	114	–	–
Median (range)	–	0 (0–0.5)	–
*Body mass index (kg/m*^*2*^*)*	95	–	–
Median (range)	–	25 (17–43)	–
*Weight (kg)*	107	–	–
Median (range)	–	67 (41–111)	–
*Tumor location*	116	–	–
Lower inner	–	2	1.7
Central	–	4	3.4
Upper inner	–	11	9.5
Upper outer	–	57	49.1
Lower outer	–	8	6.9
Other	–	34	29.3
*Heart volume supine (mL)*	116	–	–
Median (range)	–	461 (259–687)	–
*Left breast volume supine (mL)*	–	–	–
Median (range)	–	484 (34–1580)	–
*Couch type*	116	–	–
Bionix (Bionix Development Corporation, Toledo, OH, USA)	–	99	85.3
Varian (Varian Biomedical Systems, Palo Alto, CA, USA)	–	17	14.7
*Patient’s preference*	78	–	–
Supine	–	41	52.6
No preference	–	14	17.9
Prone	–	23	29.5
*Treatment applied*	116	–	–
Supine	–	71	61.2
Prone	–	45	38.8

*N* number with the data available

**Table 2:** Robust linear regression analysis of the penalty difference prone − supine

Characteristics	Intercept	Coefficient	SD	Range prone better	Unit
PRE-DOSIMETRY					
*Pendulousness*					
Breast depth ratio prone/supine^a^	1.244	−0.7950	**0.2493**	**>1.6**	**Ratio**
Breast ∆ depth difference prone − supine^a^	0.338	−0.0111	**0.0033**	**>31**	**mm**
Breast depth prone	0.444	−0.0057	**0.0020**	**>77**	**mm**
*Breast/body*					
Breast volume/body weight ratio	0.278	−0.0564	**0.0273**	**>4.9**	**mL/kg**
*Tumor location*					
Lower inner quadrant (LIQ) vs. not LIQ	−0.176	2.7829	**0.5075**	**Not LIQ**	**Binary**
*Breast size*					
Left breast volume supine	0.207	−0.0007	**0.0003**	**>282**	**mL**
Right breast volume supine^a^	0.177	−0.0005	**0.0002**	**>347**	**mL**
Breast depth supine	0.278	−0.0073	0.0038	>38	mm
*Body size*					
Weight	0.474	−0.0087	0.0046	>54	kg
Body mass index	0.156	−0.0115	0.0143	>14	kg/m^2^
Heart volume supine	0.217	−0.0008	0.0008	>263	mL
Height	3.029	−0.0193	0.0118	>157	cm
*Deep inspiration breath-hold (DiBH) capability*					
Right lung volume supine	−0.708	0.2071	0.1484	<3.4	L
Total lung volume supine	−0.640	0.0970	0.0766	<6.6	L
Left lung volume supine	−0.537	0.1641	0.1542	<3.3	L
Age	0.261	−0.0074	0.0064	>35	Years
LAD–chest wall distance	−0.005	−0.0056	0.0102	NI	mm
*Other*					
Couch type Varian vs. Bionix	−0.124	−0.2865	0.1868	NI	Binary
Preference prone vs. else	−0.130	−0.0437	0.1785	NI	Binary
*POST-DOSIMETRY*					
Prone penalty score	−2.134	0.8961	0.0389	<2.4	%dose
Supine penalty score	1.464	−0.7004	0.1008	>2.1	%dose

Significant characteristics (|coefficient/SD| >1.96) are highlighted in bold

*SD* standard deviation of the coefficients, *NI*: cutoff not identifiable

^a^The cutoffs using nonlinear transforms were as follows: ratio: 1.7; ∆ depth: 35 mm; right breast volume: 543 mL

The models allow the extraction of the range of values for which a dosimetric gain with the prone setup might be predicted. Dividing the intercept with the coefficient provides the cutoff where the gain changes sign. Table 2 shows a reduction in the prone penalty at a breast depth ratio prone/supine >1.6, breast ∆ depth >31 mm, breast depth prone >77 mm, left breast volume >282 mL, and right breast volume >347 mL. Regression excluding the three outliers showed a loss of significance in the characteristics of breast/body weight and lower inner quadrant, in line with the plots (Fig. 6b). The cutoffs computed without outliers were of comparable magnitude, depth ratio >1.5, ∆ depth >23 mm, depth prone >62 mm, breast volume left >197 mL, and right >193 mL.

The post-dosimetry penalty scores were significant predictors of the dosimetric gain (Table 2). These simulate the situation of only one treatment plan when either the prone or the supine setup is available. If the prone penalty score is already <2.4, changing to the supine setup is unlikely to further reduce the score. Conversely, if the supine penalty score is >2.1, changing to the prone setup has a good likelihood of reducing the penalty, as inferred earlier from the inspection of the bullet-arrow chart (Fig. 5).

## Discussion

Dosimetric gain analysis in this patient population that included breast volumes as small as 34 mL and two breast implant patients demonstrated a preponderant advantage of the prone setup as compared with the supine setup. The benefit of the prone setup was observed in 62.1% of the cases.

Our results confirm the general strong benefit of prone positioning on lung dose [29], which was reduced from 7.6% of the prescribed dose in supine position to 1.9% in prone, and the benefit of DiBH with regard to the mean heart dose [22, 23], which was reduced from 3.4% prone to 1.9% supine.

The idea to predict the benefit of prone position instead of dual planning is not new. Zhao et al.’s support vector machine selected heart orientation, heart–tumor distance, and in-field lung volume as the best features to reduce the proportion of prone-treated patients who would have required a second supine CT [51]. They drew attention to the (unmet) need to determine the plasticity of deformation and displacement of organs between the two positions. Lymberis et al. reported that 46/53 (87%) cases of left breast cancer were best treated prone, in all with breast volumes >1500 cc, in 95% with 750–1500 cc, and in 68% with <750 cc [52]. In a study of 138 left breast cancers, Varga et al. established a model based on BMI, median distance between LAD and the chest wall, and heart area included in the radiation field on a single supine CT slice as the most appropriate predictor for the choice of positioning [31]. Kahan et al. confirmed the utility of the model, the treatment position was prone in 67/100 (67%) and 47/60 (78.3%) patients of a validation and a clinical practice series, respectively [53]. Rarosi et al. analyzed the predictive performance of different statistical models of BMI, LAD distance, and in-field heart area on the LAD mean dose difference between supine and prone position [54]. Multiple linear regression appeared to be the most useful model.

The present study is novel as the previous benefit predictions compared prone setup to a free breathing, not a DiBH supine setup. Our approach also differs. We defined a penalty score taking into account all structures, reducing the question of a benefit to a single number which is easy to compare. Furthermore, in some previous publications only heart doses were considered although dose sparing in the lung can indeed be very important [55].

However, the approach has drawbacks. The “type 1” weights of heart, lungs, breasts, and tumor bed reflected a single institution’s choice. Weights may need to be defined according to probability and severeness of side effects in the different targets or organs at risk. The importance of lung, heart, and breast dose is not the same for each patient. To select patients for prone or supine setup, individual risks need to be considered [56].

These are key issues. Therefore, we expand the analysis in this discussion. Instead of the “type 1” penalty score, alternative organ-specific (or side effect-specific) scores were evaluated. Table 3 lists alternative sets of weights according to four broad categories of patients’ clinical conditions. The *heart* priority type would represent patients with history of heart disease, presenting cardiovascular comorbidity, or receiving cardiotoxic therapy such as doxorubicin or trastuzumab, thus requiring most heart protection [27]. The *lungs* priority type would apply to patients with higher age or a history of pulmonary disease [9, 57]. The *PTVs* priority type would be patients at a high risk of local relapse, such as large tumors or negative hormone receptor status [58]. The *body* priority type might be patients in whom radiation-induced cancer is a concern, for whom precedence might be given to avoid irradiation of large volumes of tissues [25, 59, 60] as well as avoiding high doses [61]. The DVH of the whole-length CT body structure, or external contour, was exported with the other DVHs. The body structure includes organs and PTVs. Using it in a penalty score would doubly penalize the OARs and PTVs. Hence its 0 weight in the preceding priority types; but here the body structure is relevant to assess how the risk of second cancer can affect the role of the prone setup.

**Table 3:** Alternative sets of penalty weights

Priority type	Weights						
	Heart	Right lung	Left lung	Tumor bed	Left breast	Right breast	Body
Type 1	*0.40*	*0.16*	*0.14*	0.11	0.09	0.10	0
Heart	*0.65*	0.08	0.07	0.10	0.05	0.05	0
Lungs	0.20	*0.30*	*0.35*	0.07	0.04	0.04	0
PTVs	0.15	0.08	0.07	*0.35*	*0.30*	0.05	0
Body	0.10	*0.15*	*0.15*	0.08	0.07	*0.15*	*0.30*

*PTVs* planning target volumes

Italicized values highlight which organ or group of organs receive the highest weights according to the priority type; in each row the sum of these values equals at least 0.65

Table 4 summarizes the penalty scores in prone and supine computed according to the priority types defined in Table 3. As might be expected, *heart* priority increases prone penalty (∆penalty = 0.41), while *lungs* priority decreases prone penalty (∆penalty = −1.64). Prone and supine are balanced with *PTVs* priority (∆penalty = 0.01). Prone penalty decreases with *body* priority (∆penalty = −0.63), which suggests that prone might have a lower risk of second cancer as compared with supine. That latter observation brings to the forefront an earlier phantom dose measurement study that called attention to the larger doses received by far distant organs—notably the eyes, ovaries, cervical, thoracic, and lumbar vertebrae—with supine tangential fields as compared with prone tangential fields [62]. Other authors also observed that the dose to nontarget areas (hotspots outside the breast PTV and other than lung or heart, such as the latissimus dorsi) was consistently reduced in the prone position [63, 64].

**Table 4:** Penalty scores according to setup and type of priority

Priority type	Penalty score Median (range)		∆penalty prone − supine Median (range)
	Supine	Prone	
Type 1	2.36 (1.33–4.26)	2.14 (0.98–10.78)	−0.21 (−2.47–7.24)
Heart	2.11 (1.26–3.89)	2.41 (1.17–14.13)	0.41 (−1.78–11.3)
Lungs	3.39 (1.37–5.91)	1.66 (0.68–8.01)	−1.64 (−4.26–3.43)
PTVs	2.19 (1.37–4.92)	2.19 (1.05–10.16)	0.01 (−3.25–7.19)
Body	3.79 (2.07–6.33)	3.08 (1.57–7.66)	−0.63 (−3.29–3.41)

*PTV* planning target volume. Penalty score’s units as percent of prescribed dose

∆penalty computed from all individual penalty prone minus penalty supine

**Fig. 7:**
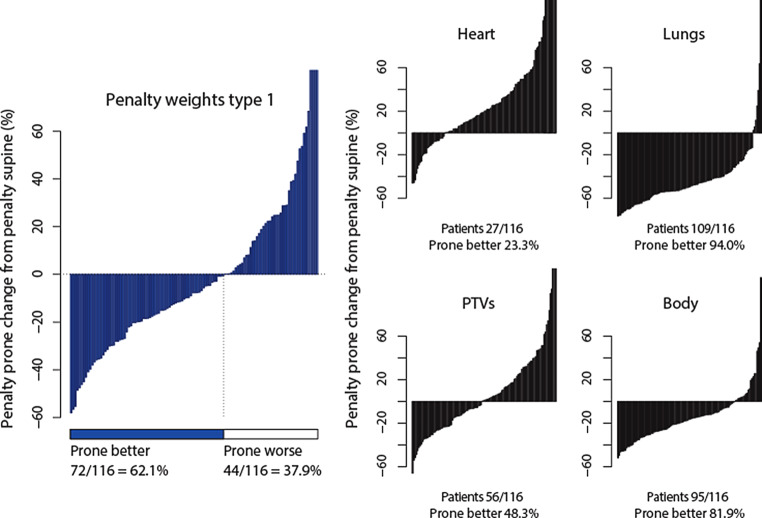
Prone penalty percent change from supine penalty, according to priority type. *PTVs* planning target volumes. Priority types defined in Table 3

Fig. 7 compares the prone penalty percent change relative to supine penalty according to the priority types. The number of patients in whom prone reduced the penalty ranged from 27 to 109 of 116. The proportion of patients was lowest but still substantial with *heart* priority, 23.3% (95% CI: 15.9%, 32.0%) and highest with *lungs* priority, 94.0% (95% CI: 88.0%, 97.5%). The type 1 priority appeared to be a fair representative average.

The outcomes show that the penalty score can be transparently adapted. Different penalty types hint at the possibility to tailor radiotherapy prescription according to a patient’s pathology. Regression might find different predictors but would best be investigated in a multi-institutional study. For the remainder of the discussion, we focus only on the type 1 priority.

Pendulousness or plasticity were the foremost predictors of a dosimetric gain (reduction of penalty score priority type 1). The breast air-to-surface ratio has been proposed as a measure of pendulousness for use with automatic segmentation [65]. The present study proposes other measures—the breast depth prone/supine ratio and breast prone − supine ∆ depth difference [66]. Both can be assessed clinically. Examining the patient lying supine followed by a standing position and leaning forward 90º might provide an indication of how much the breast sags from the chest wall, thus helping to select the most favorable position for the planning CT. Among the 3 patients with extreme prone penalty scores (cases 187, 136, and 11, Fig. 5), the breast depth ratios were 1.3, 1.5, and 1.2, and the breast ∆ depths were 15, 19, and 11 mm, respectively. Case 11 had a breast implant, case 136 had a lower inner quadrant tumor, and case 187 had a scar that appeared to hinder the breast’s displacement (Fig. 1). Applying the pendulousness assessment just mentioned, they would most likely not have been selected for a prone setup.

Several pitfalls are worth underscoring. Indeed, the mean absolute dose deviation is a new metric that has been conceived to implement the present analysis [43]. Weighted penalty scoring and dosimetric gain analyses are uncharted territories. We have already expanded on the impact of weights on the gains. Choosing penalty score weightings may require a multi-institutional consensus if the present methodology has to be widely adopted in the future. The treatment delivery relied on 3D dose distributions which were not reviewed. A dose distribution assessment of the LAD, either prone or supine, was dismissed as it was not part of our standard contouring policy at the time the study was undertaken. The study was retrospective and subject to selection biases. Few women were at the lower and higher ranges of the breast volume and weights, thus potentially yielding unreliable cutoff estimates. The analyses were not designed for validation.

Strengths are also worth mentioning, most especially the perspective of a new dosimetric paradigm. The MADDs can be extracted from dose–volume histograms; dosimetric data can be analyzed even when the original treatment planning files are no longer available. The penalty scores provide a quantitative parameter to streamline the ranking of treatment plans. The weights can be adapted to the patient’s pathology as discussed earlier (Tables 3and 4, Fig. 7). Although the LAD dose distribution was not available, it has been shown to correlate with the mean heart dose, with *R* = 0.87 in both setups [54]. Moreover, our “priority type 1” penalty score gave the highest priority to the heart. Every patient underwent dual CT planning, providing a good balance of patients’ characteristics to compare prone with supine setups. Although data were collected retrospectively, all treatment plans were created prospectively, aiming to select the best setup in an individualized manner.

Last but not least, any treatment decision implies trade-offs between organs at risk and target coverage [18]. As recently suggested by other research groups, implementing DiBH in prone setup conditions might represent an even better solution, potentially providing the best for both heart and lung sparing [67, 68]. Another improvement might be the use of breast cups for prone [26, 69], which one of the authors is currently investigating. While waiting for validation studies of new techniques, the present results can help to better select patients for prone or supine treatment.

## Conclusion

A dosimetric gain was associated with a prone setup in 62.1% of patients. Measures of breast pendulousness or plasticity, breast depth ratio, or breast depth difference were the most significant predictors of gain. Pending a prospective evaluation, these measures might help to identify which treatment position, either prone or supine, could be most advantageous.
